# Biodegradation of Different Types of Plastics by *Tenebrio molitor* Insect

**DOI:** 10.3390/polym13203508

**Published:** 2021-10-13

**Authors:** Piotr Bulak, Kinga Proc, Anna Pytlak, Andrzej Puszka, Barbara Gawdzik, Andrzej Bieganowski

**Affiliations:** 1 Institute of Agrophysics, Polish Academy of Sciences, Doświadczalna 4, 20-290 Lublin, Poland; k.proc@ipan.lublin.pl (K.P.); a.pytlak@ipan.lublin.pl (A.P.); a.bieganowski@ipan.lublin.pl (A.B.); 2 Department of Polymer Chemistry, Institute of Chemical Sciences, Faculty of Chemistry, Maria Curie-Sklodowska University in Lublin, Gliniana 33, 20-614 Lublin, Poland; andrzej.puszka@umcs.pl (A.P.); barbara.gawdzik@poczta.umcs.lublin.pl (B.G.)

**Keywords:** mealworm, waste management, entomoremediation, bioremediation

## Abstract

Looking for new, sustainable ways to utilize plastics is still a very pertinent topic considering the amount of plastics produced in the world. One of the newest and intriguing possibility is the use of insects in biodegradation of plastics, which can be named entomoremediation. The aim of this work was to demonstrate the ability of the insect *Tenebrio molitor* to biodegrade different, real plastic waste. The types of plastic waste used were: remains of thermal building insulation polystyrene foam (PS), two types of polyurethane (kitchen sponge as PU1 and commercial thermal insulation foam as PU2), and polyethylene foam (PE), which has been used as packaging material. After 58 days, the efficiency of mass reduction for all of the investigated plastics was 46.5%, 41.0%, 53.2%, and 69.7% for PS, PU1, PU2, and PE, respectively (with a dose of 0.0052 g of each plastic per 1 mealworm larvae). Both larvae and imago were active plastic eaters. However, in order to shorten the duration of the experiment and increase the specific consumption rate, the two forms of the insect should not be combined together in one container.

## 1. Introduction

According to available data from 2019, worldwide plastic production reached 368 Mt (mega tons). The largest amount of plastic was produced in Asia—51% (of which China accounted for 31%), followed by NAFTA countries (North America, Canada, Mexico) 19%, Europe 16%, Middle East, and Africa 7%, Latin America 4%, and Commonwealth of Independent States (CIS) countries 3% [1]. In Europe, of the 29.1 Mt of plastic waste collected in 2018, 32.5% was recycled, 42.6% went to energy recovery, and 24.9% to landfills [1]. Although, as compared to 2016, in 2018 the annual average for recycling and energy recovery of plastic waste increased in Europe by 5.7% and 4.8%, respectively, a significant proportion (24.9%) still end its life cycle in landfills [1]. Furthermore, much of the plastic escapes waste collection systems, polluting the environment and entering the world’s oceans. It was estimated that, e.g., in 2010, that of the 275 Mt of plastic produced, as much as 4.8 to 12.7 Mt ended up in the aquatic environment [2]. Adequate waste management policies, as well as the improvement of the plastic waste management methods used, have a huge impact on the environment. In order to reduce the onerous problem of plastic waste, the European Commission has presented a plan which includes, among other things, allowing only reusable or recyclable plastic on the market and recycling at least half the amount of plastic produced [3].

Currently, various forms of recycling are used. This includes mechanical recycling, which involves re-introducing plastic waste into the production cycle and plasticizing it. All thermoplastics such as: polyethylene (PE), polypropylene (PP), and polystyrene (PS) are suitable for this procedure due to their ability to change shape under high temperature conditions. The disadvantage of this method is that plastics must be carefully sorted before recycling. However, it should be remembered that the food industry uses packaging of a high standard, so in most cases, recovered plastics must not be allowed to come into contact with food. Recycled materials usually go into the production of items of lower value (downcycling), such as garbage bags and traffic cones [4]. 

Chemical recycling includes activities connected with converting plastic waste into valuable chemical substances or into precursors of plastic. One of the ways can be by pyrolysis, where in anaerobic conditions under the influence of high temperatures and a catalyst plastics decompose, and which contributes to the production of biofuel [5]. Another chemical method is the gasification of plastics, the conversion of waste into gaseous products which can be used in the energy industry (it is necessary to control the level of chlorine content during this process) [6]. 

Another alternative way to reduce residual waste is to produce plastics capable of degradation under certain natural conditions, such as exposure to sunlight, moisture, having shorter composting times or types of plastics that can be degraded by living organisms. These plastics are called bioplastics, and include polyhydroxyalkanoates (PHA) and polylactide (PLA). Plastics made of petroleum-based polymers, such as PE, PP, PS are difficult to biodegrade. It was long thought that they are not available as carbon source for microbiota. Fortunately, it was found that some microorganisms possess the ability to produce enzymes that break down all these types of plastics [7,8]. The course of biodegradation is influenced by factors, such as the characteristics of the polymer itself (type of chemical bonds) and the storage conditions of the plastic waste. Biodegradation is highly anticipated and future-oriented natural method of plastic waste disposal [9].

There are many studies which show that some bacteria and fungi, especially those associated with the soil, are promising organisms for the biodegradation of plastic waste. The numerous examples include, e.g., the bacteria *Brevibacillus borstelensis* [10], *Bacillus brevis* [11], *Pseudomonas stutzeri* [12], and the fungi *Rhizopus delemar* [13], *Mucor* sp., *Paecilomyces* sp., and *Thermomyces* sp., [14], although more specific research is still needed on all of these, especially in order to increase their biodegradation potential.

Biodegradation potential has also been seen in more complex organisms, such as insects. *T. molitor* has the potential to consume PE, PS, PP, polyurethane (PU), polylactic acid (PLA), and polyvinyl chloride (PVC) [15,16,17,18,19,20,21] and even tire crumbs and vulcanized butadiene-styrene elastomer (SBR) rubber [22]. The biodegradability of PE and PS is provided by the larvae of the beetle *Tribolium confusum* [23] and the superworm *Zophobas atratus* [21]. Another example is *Galleria mellonella*, a wax moth, which is capable of PE degradation [24]. Studies have shown that the ability of the insect to biodegrade plastics is in most cases dependent on its intestinal microorganisms. For example, the fungus *Aspergillus flavus* contributed to PE degradation in the abovementioned wax moth [25]. However, studies on *Corcyra cephalonica* rice moth larvae have proven that they have the ability to degrade low density PE (LDPE) even after antibiotic therapy, indicating that biodegradation is not dependent on the activity of intestinal microflora enzymes [26]. These are examples of entomoremediation [27,28] of plastics, a new subtype of bioremediation.

The recent discovery of the land snail *Achatina fulica,* able to disintegrate PS [29] regardless of the composition of the intestinal microflora, in our opinion is an outstanding example of how little we know about the enormous potential hidden in nature when it comes to the utilization of plastics.

*T. molitor* is a species of beetles from the Tenebrionidae family, found in temperate climate regions and considered a storage pest. *T. molitor* larvae is yellow, which is why it is commonly called a “yellow mealworm”. Its size varies between 2.5 and 3.5 cm. The adult form is black and measures approximately 1.5 cm [30]. Mealworm larvae are full of nutritional value, making them suitable as live animal feed, e.g., for the zoo industry [31,32]. They are also considered an alternative source of protein in the food industry [33,34]. *T. molitor* frass can be used as a substitute or additive for NPK fertilizers [35], but it can also be used to produce biogas through anaerobic digestion [36]. *T. molitor* itself is likely to have probiotic properties. Studies have shown that adding them to the diet of mice results in the growth of bacteria from the *Bifidobacteriaceae* and *Lactobacillaceae* families in their intestines [37]. Another study has found the enrichment of the beneficial intestinal microflora in rabbits, as well as a reduction in pathogens in their digestive tract [38]. *T. molitor* can also be a source of chitosan production, which can be used to produce UV-protective packaging due to its naturally opaque, brownish color [39].

The aim of the study was, therefore, test the performance of biodegradation of real plastic wastes by *T. molitor*, including polystyrene (PS), two types of polyurethane (PU) and polyethylene (PE) and, for the first time, provided data about the efficiency of polyurethane biodegradation by mealworms. To demonstrate how the process of plastics biodegradation by *T. molitor* really looks like we created time-lapse video. We measured also growth parameters of the insects in order to show how they performed on the wastes and discussed advantages and disadvantages of used approach. Heavy metals and other elements in plastics were also analyzed to see how their content affected the disposal of plastics by mealworms.

## 2. Materials and Methods

### 2.1. Insect Rearing

*T. molitor* larvae were ordered from an external supplier. The larvae were fed with wheat bran (KUPIEC, Poland). The dry weight (DW) of the bran was 87.92 ± 0.01% (105 °C/24 h) and pH_H2O_ = 6.56 ± 0.04 (*w*/*v* 1:20). The mealworms were reared in the following conditions: temperature 24 ± 1 °C, relative humidity 60 ± 5%, in plastic containers at the Department of Natural Environment Biogeochemistry, Institute of Agrophysics PAS, Lublin, Poland. After the acclimatization of the larvae (3 d) they were sifted from wheat brans and left for 24 h to empty their intestines.

### 2.2. Plastic Waste

Four types of plastics were tested in the experiment:

1. Polystyrene (PS) in the form of Styrofoam, which is used for insulating building elevations;
2. Polyurethane foam (PU1)—in the form of kitchen sponges;
3. Polyurethane thermal insulation foam (PU2)—consisting of polyphenylpolyisocyanate polymethylene, according to information from the producer (SOUDAL Sp. z.o.o, Poland);
4. Polyethylene foam (PE)—which is used as filling material in packages, e.g., to protect electrical equipment during transport.

**Figure 1:**
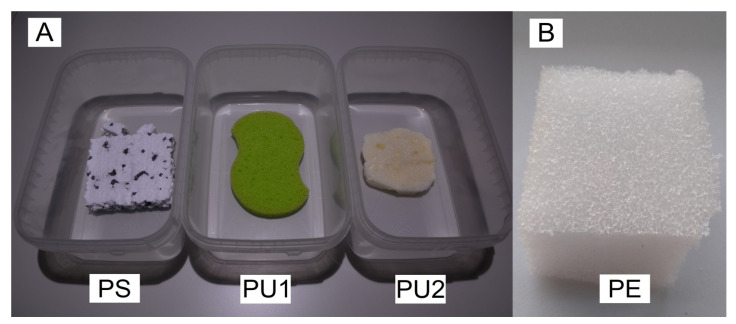
Plastics used for the experiment. (**A**) Materials for which time-lapse video was created. From left: polystyrene (PS), polyurethane kitchen sponge (PU1), polyurethane thermal insulation foam (PU2). (**B**) Polyethylene packaging foam (PE).

The plastic used is showed on Figure 1. Kitchen sponge (PU1) was used without the abrasive layer, which is often added to that type of houseware item. A day before the beginning of the experiment, PU2 was removed from the original packaging and left to bind in the form of one large mass. The following day, a thick slice was cut from the inside of this piece and was used in the experiment. All plastics were cut to obtain an equal weight of 2.6 g (±0.001 g) before the experiment begun, i.e., to match to the weight of the PU1 (kitchen sponge), which was taken as a whole

The original plan of the experiment assumed the use of only PS and two types of PU, but during the course of the experiment, we decided to add one more variant with PE foam, as there was no research on the possibility of PE foam biodegradation by mealworm larvae at that time. However, adding the PE variant was not possible in the experiment, which was just being filmed, so the time-lapse video shows only 3 types of plastic.

### 2.3. Experimental Procedure

Each of the four experimental variants was carried out in three independent replications in plastic boxes with dimensions of 30 × 19 × 12 cm (see Figure 1). The experiment was conducted in a laboratory room under controlled conditions: temperature 24 ± 1 °C and relative humidity 60 ± 5%. Each container contained 500 mealworm larvae with a length and weight of individual larvae about 2.39 ± 0.02 cm and 0.100 ± 0.010 g, respectively. All variants (PS, PU1, PU2, PE) included 2.6 g of a given plastic, therefore at the beginning of the experiment 0.0052 g of each plastic was available per 1 mealworm larvae (based on literature data and our preliminary trials). The substrates were not enriched with additional substances such as water or feed. 

The substrates were weighed daily on a laboratory balance (OHAUS EX224M, Parsippany, NJ, USA) in order to investigate the loss of plastic. During the experiment, when the pupae started to appear massively, they were taken out of the container with tweezers and put into a separate glass vessel for pupation. After completion of this process, the adult beetles were once again moved back into the original container. This operation can be seen on the recorded Video S1. Adult beetles are also capable of eating plastics and can survive on this substrate as the sole source of food, similar to the larvae. As mealworm pupae are not mobile, removing the pupae during the experiment was performed in order to protect them from cannibalism by other developmental stages of the insect. Cannibalism, which is always present in mealworm breeding, would reduce the amount of eaten plastic.

The experiment lasted 58 days and was completed when the consumption of plastics by the insects became minimal. After that time the weight of the insects was determined by the use of a laboratory balance and their length using a hand ruler. In order to separate insect excrement from the uneaten plastic remains, they were sieved through a 500 µm mesh size sieve. 

The utilization rate (U) of the plastics was calculated based on the following Equation (1):

(1) $$U=\frac{m_{i}-m_{f}}{m_{i}}\cdot 100\%$$

where:

*m_i_*—initial mass of plastic (i.e., at the beginning of the experiment) (g);

*m_f_*—final mass of plastic (i.e., at the end of the experiment) (g).

Plastic remnants that passed through insect digestive tract were extracted from the mealworm frass collected after the experiment. One gram of the frass from each experimental variants was demineralized with 5% HCl by 1 h in room temperature and then digested using 30% H_2_O_2_ by 48 h in room temperature. Digestates were filtered on glass filter with pore size 1 µm [40]. After that the samples were dried in 50 °C for two days (until no mass changes were noticed).

### 2.4. Fourier Transform Infrared Spectroscopy (FT-IR) of Plastics

**Figure 2:**
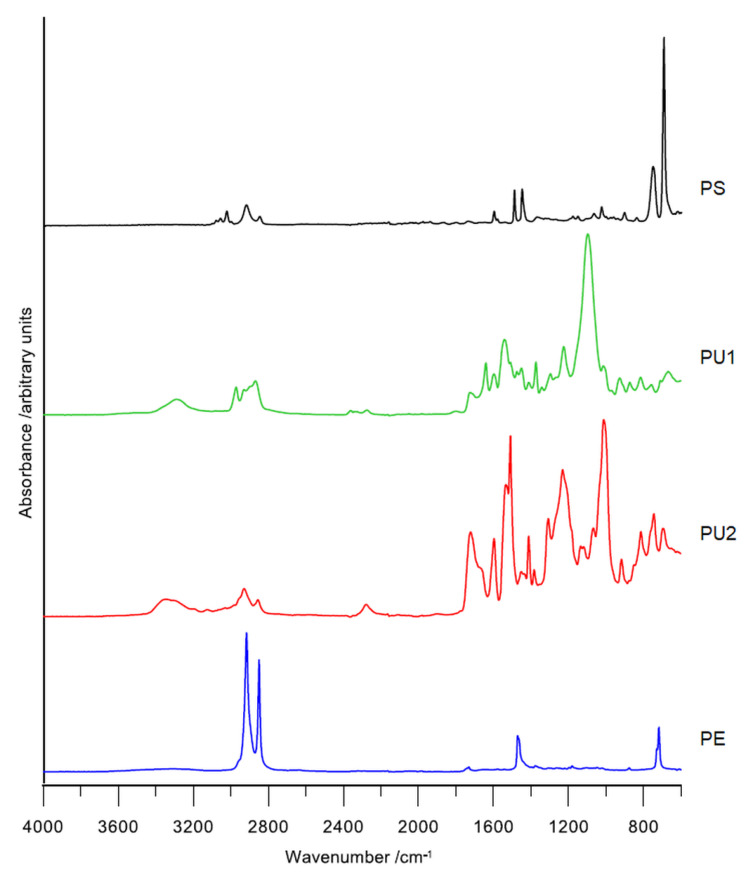
FT-IR spectra for plastics used in the experiments. PS—polystyrene (Styrofoam), PU1—polyurethane foam (kitchen sponge), PU2—polyurethane foam (building thermal insulator), PE—polyethylene foam (packaging foam).

The FT-IR spectra were developed by applying attenuated total (internal) reflection (ATR/FT-IR) with the use of a FT-IR TENSOR 27 spectrophotometer (Bruker, Germany), complete with a *PIKE* measuring cell which features crystalline diamond embedded in zinc selenide. The FT-IR spectra were collected within the range of 4000 to 600 cm^−1^, with 32 scans per sample, at a resolution of 4 cm^−1^. The absorption mode was used for these measurements. Figure 2 showed spectra obtained for used materials.

The FT-IR spectra of the tested plastics are shown in Figure 2. The peaks at 2921cm^−1^ and 2850 cm^−1^ correspond to C–H asymmetric and symmetric stretching in CH_2_ groups. The presence of a benzene ring in PS is confirmed by absorption bands at 3060 cm^−1^ and 3026 cm^−1^ (correspond to C–H stretching in benzene ring), band at 754 cm^−1^ corresponds to out-of-plane C–H bending of the benzene ring. Absorption peaks at 1601 cm^−1^, 1493 cm^−1^, 1452 cm^−1^ are related to C=C stretching of the benzene ring. The absorption band at 696 cm^−1^ with high intensity corresponds to a monosubstituted benzene ring.

The FT-IR spectra of both polyurethane materials show characteristic absorption bands responsible for N–H stretching vibrations (in the range 3290 to 3340 cm^−1^), N–H bending (at 1509 cm^−1^) and C=O stretching (in the range 1727 to 1721 cm^−1^) of the urethane group and absorption bands at 2929 cm^−1^ and 2868 to 2855 cm^−1^ (asymmetrical and symmetrical, respectively) corresponding to C–H stretching vibrations of the CH_2_ group, and at 1383–1373 cm^−1^ corresponding to C–C bending vibrations.

The spectra of PE show characteristic absorption bands at around 2915 cm^−1^ and 2849 cm^−1^ connected to C–H stretching in the CH_2_ group while absorption band at 1471 cm^−1^ correspond to C–H bending in CH_2_ group. Absorption bands at 729 cm^−1^ and 718 cm^−1^ are connected to C–H bending in-plane in CH_2_ groups.

### 2.5. Energy Dispersive X-ray Fluorescence (EDXRF)

The concentrations of elements in the plastics samples were measured by means of EDXRF using EDX-7000 (Shimadzu, Kioto, Japan). Measurements were completed in air atmosphere using default plastics measuring program with autobalance. Measurement time was 100 s on each channel and collimator had 10 mm ϕ. Samples were placed directly in the Rh X-ray beam without the use of foil or special containers. 

### 2.6. Scanning Electron Microscopy (SEM)

Plastics samples taken for the experiment, as well as plastics remnants isolated from 

the insect frass after the experiment were sputter coated with a 30 nm Au layer in the coater EM ACE (Leica, Germany) and visualized by the use of Libra SEM (Carl Zeiss, Germany) set to the following parameters: work distance (WD) 9–15 mm; spot size 360–370, accelerating voltage 5 keV; aperture size 30 µm; beam current 30 µA; signal SE detector with scanning mode and pixel noise reduction.

### 2.7. Photography and Time-Lapse Movie of Utilization

Photography was completed with the use of a Nikon D7100 with an AF-D DX NIKKOR 18-105mm f/3.5-5.6G ED VR lens. Photographs were taken automatically every 30 min and a time-lapse movie was created from them using Adobe After Effects 2020 and Adobe Media Encoder 2020.

### 2.8. Statistical Analysis

Experimental results were analyzed using Statistica 13.1. The statistical significance was determined by t-Student test and ANOVA with post hoc Tukey’s test (*p* < 0.05; n = 3). Three independent biological replications of the experiment were performed.

## 3. Results

### 3.1. Rate of PS, PU1, PU2, and PE Consumption by Mealworms

**Figure 3:**
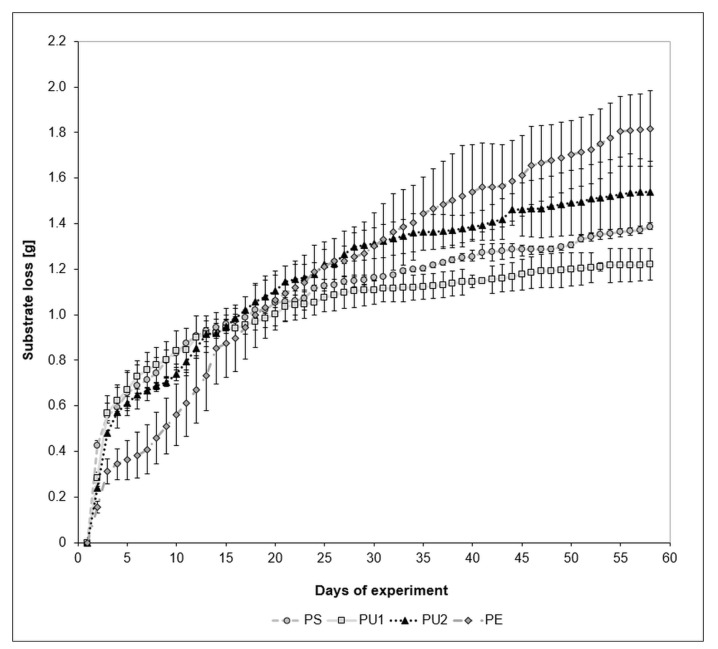
Cumulative loss of PS, PU1, PU2, and PE plastic waste (g) during feeding of *T. molitor* (mean ± SD; n = 3).

Figure 3 illustrates the weight reduction in plastics by *T. molitor* insect. The first significant (Student *t*-test, *p* < 0.05) loss of mass for each type of plastic was observed on the third day of the experiment and amounted to 0.566, 0.569, 0.482, and 0.313 g (PS, PU1, PU2, and PE, respectively). On the 15th day, the most uniform utilization values of individual plastics were observed, ranging from 0.85 to 0.94 g. On the 58th day of the experiment, statistically significant differences in the reduction mass of the plastics were shown and were as follows: PS 1.385 g, PU1 1.221 g, PU2 1.538 g, and PE 1.818 g, which is equivalent to a percentage loss of 46.93 ± 0.12%, 46.77 ± 2.73%, 58.97 ± 5.15%, and 69.71 ± 6.34%, respectively (Table 1) (PE > PU2 > PS > PU1). Interestingly, at the beginning, the utilization of PE was the lowest, however, it was ultimately the highest of all the plastics used in this trial.

### 3.2. Morphological Parameters of Mealworm Larvae

Table 2 shows morphological parameters of *T. molitor* larvae. At the end of the experiment, the mass of 1 larvae in all variants, i.e., PS, PU1, PU2, PE decreased by 18.37%, 28.28%, 26.26%, and 24.71%, respectively. Similarly, decrease in larvae length was also observed and amounted for PS 6.26%, for PU1 9.35%, for PU2 4.35%, and for PE 6.58% (Table 1). 

### 3.3. Elements Content in the Plastics

Investigated plastics had in general trace amounts of different elements (Table 3), with some exceptions. PS had the highest concentration of Br, amounted to 0.412 ± 0.019%. The samples of polyurethane kitchen sponge (PU1) was characterized by high concentration of Ca (10.110 ± 0.301%), Al (0.437 ± 0.038%), Si (0.358 ± 0.027%), and Cl (0.224 ± 0.022%). The second investigated polyurethane material (PU2) had different elemental composition and characterized by high content of Cl (7.574 ± 0.141%) and P (1.379 ± 0.035%). PE had only minor content of Al (0.180 ± 0.029%) and Ca (1.381 ± 0.020%). Interestingly in PS and PE trace amounts of Hf was detected.

### 3.4. Scanning Electron Microphotography of the Plastics Surfaces

**Figure 4:**
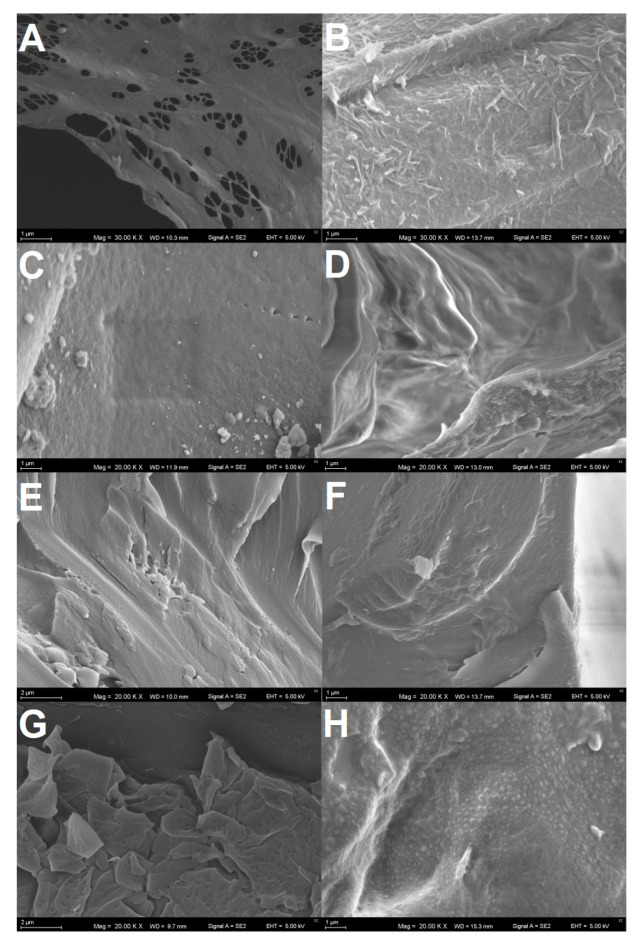
SEM microphotography of the plastic samples taken for the experiment (**A**,**C**,**E**,**G**) and plastics isolated from the insect frass after the experiment (**B**,**D**,**F**,**H**). (**A**,**B**): PS (30,000×); (**C**,**D**): PU1 (20,000×); (**E**,**F**): PU2 (20,000×), (**G**,**H**): PE (20,000×). Magnifications were selected to best illustrate the differences in the surface of the samples.

Figure 4 showed SEM microphotography taken from the plastic samples, which were used in the experiment and those which were isolated from the insect frass. At the beginning all materials were characterized by smooth surfaces. PS (Figure 4A) exhibited a smooth and delicately cross-linked surface structure with visible longitudinal cracks arranged in the same direction which were most likely caused by mechanical impact during sample preparation (breaking and cutting of brittle material). PS isolated from the frass showed completely different, rough and irregularly carved surface structure (Figure 4B). PUs foams (Figure 4C,E) structures were at the beginning also smooth but PU1 (Figure 4C) was more flat while PU2 (Figure 4E) showed a greater number of edges. After the passage of the digestive system of insects, the surface of the PU foams was significantly wrinkled (Figure 4D,F) and the edges clearly visible in the PU2 structure at the beginning were significantly smoothed. At the magnification of 20,000× PE surface consisted of the flat valleys and flaky layers of material superimposed on one another with visible fine pores present in a small amount (Figure 4G). After digestion by the insect PE had undergone wrinkles, the pores were no longer visible and instead of them numerous small bubble-like protrusions have appeared (Figure 4H).

### 3.5. Time-Lapse Movie of Utilization

Video S1 shows the setup used for this experiment. The initial mass of each plastic (2.6 g) was adopted due to the weight of the PU1, which was a whole unused kitchen sponge. We tried to adjust the mass of the other plastics so that they were in one piece, but it was not always possible, and some required more material to be added (as seen in the example of PS). The sharp changes in the position of the plastic pieces visible in the video resulted from weighing them during the experiment in order to obtain data for Figure 3.

## 4. Discussion

### 4.1. Rate of PS, PU1, PU2, and PE Consumption by Mealworms

The results of PS have shown a higher degree of utilization compared to the studies of [16], where the reduction was only 9.0%, and [20,41], where the reduction was 31.0% and 39.1%, respectively. Mealworms from three Chinese regions of Guangzhou, Tai’an, Shenzhen utilized 57.5%, 34.4%, and 52.4% of PS, respectively [19]. In the study [42], authors used two mealworm species (*T. molitor* and *T. obscurus*) and reached 41.5% and 55.4% of PS utilization. They also found that the application of food additives in the form of wheat bran for *T. molitor* and corn flour for *T. obscurus* resulted in increased PS utilization: 56.8% and 67.1%, respectively. A similar increase was obtained by [43], where the addition of wheat bran to PS increased the utilization rate from 31.7% PS to 54.4%.

To our best knowledge there are no reports about the efficiency of PU utilization by mealworm. The researchers [44] investigated the epigenetic modification of mitochondrial DNA in *T. molitor* caused by PU as a sole source of feed, however they did not present any utilization parameters. Others [45], applied another insect species, *Zophobas morio*, from the same family as *T. molitor* (Tenebrionidae) to utilize PU, but the degradation was only 6%. Our results showed a remarkably higher efficiency. Utilization of PU1 (kitchen sponge) and PU2 (commercial insulation foam) (Figure 3, Table 1) amounted to 46.8% and 59.0%, respectively. This significant difference between both materials was probably caused by the different chemical composition (Figure 2) and, hence, its different macrostructure and properties (e.g., hardness). PU1 was soft and elastic, while PU2 was rigid and brittle. The observations show that regardless of the material itself, it is much easier for mealworms to bite and chew something that is stiff and brittle than flexible and soft. FTIR spectra showed the presence of different additives in both PUs (Figure 2), while element content analysis (Table 3) allow to deduce to which classes of chemical compounds these additives can belong (discussion below, see Section 4.3). Moreover, FTIR spectra confirmed that PU1 was soft PU due to the presence of the 1120 cm^−1^ band characteristic for aliphatic alcoxy-groups (Figure 2). PU2 was rigid foam due to presence of the band around 1600, 100, and 650 cm^−1^, which confirmed ring structure of PU2 formulation (Figure 2). The spectrum showed also 2 characteristic bands at 1250 and 1040 cm^−1^, which indicated the aromatic alkoxy-groups (Figure 2). Soft foams are formed of polyols with a molecular weight of 2000 to 8000 units and diisocyanates, while rigid foams are made of polyols with a molecular weight of less than 1000 units and a mixture of di- and triisocyanates. Summarizing, the differences in the utilization of PU1 and PU2 resulted from the different chemical composition of both materials, as well as the difference in mechanical properties. The results obtained for PE reduction were higher than the results presented in the literature. [19] exploited the mealworm larvae for PE disposal and showed that the origin of the larvae (different regions in China) influenced the degree of utilization. The results were: 36.9% loss of PE for the larvae from Guangzhou, 22.0% for larvae taken from Tai’an, and 29.7% for larvae from Shenzhen. These differences were probably caused by the different microbiomes inhabiting the intestines of the larvae. The addition of wheat bran increased the degradation rate of PE from 48.3% to 61.1% [43].

It must be mentioned that not 100% of the eaten amount of given plastic is assimilated by the mealworms. As revealed by [20], 47.7% of ingested PS carbon was converted to CO_2_ and ca. 0.5% was assimilated into biomass (lipids). This indicated that ca. 50% of PS left in the frass [20]. Our results showed that the total amount of the plastics remnants in the mealworm frass was in the range of 31.7% to 45.4% (Table 1). PS was the most susceptible to biodegradation while PU1 the least. The relatively high presence of plastics in the feces is disadvantageous due to the possibility of spreading microplastics, however research is needed on the susceptibility of these residues to microbial degradation, as it can be changed due to the action of enzymes in the insects digestive tract.

Table 4 showed specific consumption rate given as µg plastic·day^−1^·larvae^−1^ calculated for our results, as well as for literature data. In most of the articles, the experiments were conducted for 30 days, therefore to calculate this parameter we used our data for plastic waste utilization also for that day. In general, specific consumption rate for PS as reported in the literature was within the range of 118.0 to 268.2 µg plastic·day^−1^·larvae^−1^ (Table 4) and can be enhanced by the addition of more natural and nutritious feed, such as soy protein, up to 491.0 µg plastic·day^−1^·larvae^−1^ [46]. PE consumption rates were in the lower range than PS: 102.7 to 226.6 µg plastic·day^−1^·larvae^−1^ (Table 4) and was increased to 286.5 µg plastic·day^−1^·larvae^−1^ by the addition of wheat bran [47]. Lower than reported so far consumption rates calculated for our results were the consequence of experiment design, in which both larvae and adult insects were used in one container to eat plastics. This increased the chance of cannibalism occurring. The second reason was the early appearance of pupae (which can be clearly seen in Video S1), which excluded a large number of insects from actively eating plastic during pupation. Pupae started to appear in large numbers starting from day 10. Specific consumptions rate calculated for this day fall within the range reported in the cited literature (Table 4).

Genetic differences between mealworm populations around the globe can be the third reason. As shown in [19], genetic differences can be significant even between national populations.

From our results it can be estimated how many mealworm insects (in terms of pieces and mass) would be needed to utilize 1 kg of each waste plastic in the same time as in the presented experiment (i.e., 58 days). During the experiment, 500 insects consumed 1.385 g of PS, 1.221 g of PU1, 1.537 g of PU2, and 1.818 g of PE. Approximating directly, the following number of insects would be needed: 361,011 pc. for PS, 409,500 pc. for PU1, 325,309 pc. for PU2, and 275,028 pc. for PE. The average mass of 500 pc. of larvae we used for the experiment was 56.166 g. Therefore, in terms of mass, one would need the following number of mealworms for the utilization of 1 kg of PS, PU1, PU2, PE during 58 days: 40.5 kg, 46.0 kg, 36.5 kg, and 30.9 kg, respectively. Such amounts are currently commercially available from large growers.

### 4.2. Morphological Parameters of Mealworm Larvae

Different studies reported different changes in mass. A decrease in the average weight of *T. molitor* larvae fed with PS differed greatly between the larvae from different sources and amounted to 3.31%, 21.70%, and 37.06% for Guangzhou, Tai’an, and Shenzhen, respectively [19]. Similar results were obtained by [42], where the reduction in *T. molitor* larvae weight was 8.6% on PS. However, in the study of [41], no significant changes in weight of larvae fed with PS were observed. Contrary, a weight increase of 2.5 ± 1.0% was observed by [46] for expanded PS.

Decrease in the mass of mealworm larvae fed with PE, which amounted to 1.03%, 22.10%, and 24.87% was also noticed for the abovementioned Chinese regions, respectively [19]. Ref. [46] used two types of PE: PE1, which had added pink colorant and PE2, which was without colored additives. On the first material, an 8.8 ± 2.1% increase in larval weight was observed and on the second one a decrease of 3.4 ± 1.6%. This allows to conclude that differences may at least be partially dependent on additives for plastics, such as colorants and fillers. 

Unfortunately, the cited research did not present the changes in insect length. Literature data [20] suggested that, most likely, the insects lose some of the fat tissue they had before the experiment and the hydration level of their bodies decreases significantly. This is because, in the studies, plastics were usually the only source of food and larvae had no access to water. Recently, ref. [47] showed that mealworm fed with polystyrene did indeed have a lower fat content than those who ate a conventional diet. Amazingly, they were still capable of successful pupation, which indirectly proves that digestion and assimilation must occurred.

### 4.3. Elements in the Plastics

To check whether differences in the content of elements may affect the utilization of plastics by mealworm larvae, their content was examined. Not even trace amounts of toxic heavy metals, such as Cd, Hg, or Pb, were identified in any of the samples. The majority of elements were present only in trace amounts (much below 0.1%).

Among all 13 identified elements in PS samples, only Br had non-trace concentration of 0.4%. The compound containing Br, which are added to plastics fall into class of flame retardants (FR) and green and red pigments [48,49]. The presence of Br can be connected with the addition of, e.g., hexabromocyclododecane, which is widely utilized FR in the formulations of PS [48]. Kitchen sponge (PU1) had the highest concentration of Ca. Ca in plastics serves, mainly, as a filling and reinforcement agent, which is added in the form of CaCO_3_ [48]. PU1 had also ca. 0.6% Si, which indicated that Ca may have been partially added as wollastonite (CaSiO_3_) or other Si compounds, which improved FR properties of the plastic [50]. The content of Cl in PU1 may be connected with the addition of green Cl-based pigments, chemically belonging to chlorinated/brominated phthalocyanine compounds [48]. Additionally, Cl-containing compounds, such as, e.g., Triclosan, could be added to PUs as antimicrobial agents [48]. Much higher content of Cl found in PU2 altogether with 1.4% P may be the result of using as FRs chlorinated alkyl phosphates [49], such as, e.g., tris(1-chloro-2-propyl) phosphate and tris-2-chloroethyl phosphate; such compounds are used in rigid and flexible PU foam formulations [48]. PU1 had also elevated concentration of Al. The compounds of these element are used, i.e., as stabilizers and FR but in much higher concentrations, therefore the presence of Al in PU1 was probably due to the use of Al-based fillers [48].

The presence of Ca in PE was the most likely connected with the inorganic filler added to the plastic to enhance its strength and decrease the cost of production, while low amount of Al may result from the use of compounds, such as mica or kaolin to surface stabilization of the material [48].

Analysis showed trace amount of quite exotic Hf metal in PS and PE samples. In PE it can be connected with the use of catalyst based on Hf metallocene compounds, which are widely used in the production of polyolefins [51]. In PS, it can be the results of contamination during production of this plastic by a manufacturer, which also produced polyolefins.

Figure S1 showed concentration of elements in the larvae after the experiment determined by EDXRF. Regardless of the variant, major mineral components of the larvae were K, Cl, P (>1%) and S (1–0.5%). Ca was present in concentrations of 0.1%, Zn and Fe in the order of 0.02–0.01% and Cu below 0.01%. Non-physiological elements present in the plastics probably not affected the mealworms due to its low concentrations.

### 4.4. Polymers Surface Alterations Visualized by SEM

Surface alterations of the plastics are characteristic of the aging processes occurring under the influence of microorganisms (present in the intestines of insects) or, more specifically, enzymes secreted by them. The folding of the previously smooth surface of the polymer and the formation of pitting was observed during the biodegradation of PS and PE with the bacterium *P. aeruginosa* isolated from the gut of *Zophobas morio* insect (Tenebrionidae; cousin of mealworm, known also as superworm) [52]. Surface elements mapping comparison of PS and PE before and after biodegradation showed that those changes were connected with increase in oxygen content, which suggests oxidative changes in the plastics [52]. Similar changes as observed by us in the case of PU foams had been observed by [53] after biodegradation trials in soil burial. SEM surface microphotography (Figure 4) was indirect proof that plastics eaten by the mealworm biodegraded during the action of microorganisms enzymes, as well as enzymes secreted by mealworms itself.

### 4.5. Time-Lapse Movie of Biodegradation

It can be seen that during the experiment, pupae started to appear quite quickly, especially on PU1 (Video S1). It was about 10 days after experiment had started. At the beginning, we had not planned to pull out the pupae but it became obvious that without this step the degradation of the plastics would be lowered due to the cannibalistic behavior of the larvae. This is a known problem in mealworm breeding, especially when the insects have limited access to water and a low protein content in the substrate [54]. One of the aims of this study was to reach as a high a utilization of plastics as possible, therefore we decided to take advantage of the adults’ ability to feed on plastics as well. When the amount of the adult insects increased in the containers, cannibalism started to occur with the larvae being the victim. It can be seen in the second part of the Video S1, adults formed specific “bundles” for a while with eaten larvae inside it. During the experiment dead adults were taken out the of the containers to prevent the larvae from preferring them as food. Our observations suggests also that the lifespan of the adults was much shorter than it would have been on optimal feed. Video S1 suggested that the adults’ ability to consume plastic can also be useful but they should not be combined with earlier stages within the one container to prevent cannibalism and decrease the utilization of plastics.

## 5. Conclusions

The efficiency of mass reduction for all of investigated plastics was 46.5%, 41.0%, 53.2%, and 69.7% for PS, PU1, PU2, and PE, respectively. However, specific consumption rates for each plastics was lower that calculated from literature data. This was due to the large number of pupae appearing less than two weeks after the start of the experiment and the combination of larvae with adults in one container, which resulted in cannibalistic behavior. Additionally, the used plastic waste were characterized by the addition of fillers and FRs, which may influenced consumption rates. The utilization of plastics can be increased by removing pupae from larvae and imago and by not combining adult and larval forms in one container. Both larvae and imago were active in the eating of plastics. More research is needed on different optimization approaches, which would reduce the number of insects used while maintaining process efficiency. Such optimization should be completed in order to decrease the costs of entomoremediation for larger amounts of plastics. The risk of spreading microplastics with insect feces which left after this process should also be determined in the future research.

## Figures and Tables

**Table 1:** Weight of the plastics at the beginning and end of the experiment and the mass of plastics remnants in the mealworm frass (mean ± SD; n = 3).

Parameter\Plastic Type	PS	PU1	PU2	PE
**Initial**				
**Mass of plastic [g]**	2.610 ± 0.001	2.611 ± 0.001	2.607 ± 0.001	2.608 ± 0.001
**Final**				
**Mass of plastic [g]**	1.225 ± 0.004 ^b,c,^*	1.390 ± 0.072 ^c,^*	1.070 ± 0.133 ^a,b,^*	0.790 ± 0.166 ^a,^*
**Utilization [*w/w* %]**	46.929 ± 0.124 ^a,b^	46.771 ± 2.734 ^a^	58.972 ± 5.146 ^c^	69.707 ± 6.341 ^c^
**Plastic remnants in frass [*w/w* %]**	31.697 ± 0.647 ^a^	45.353 ± 1.548 ^d^	41.724 ± 0.543 ^c^	38.977 ± 1.435 ^b^

Significant differences between initial and final values of a given parameter are indicated with * (Student *t*-test, *p* < 0.05). Post hoc Tukey’s test was completed to show significant differences between all the plastics within a given variable (different letters; *p* < 0.05).

**Table 2:** Average length and fresh weight of the insects at the beginning and end of the experiment (±SD; n = 3).

Parameter\Substrate	PS	PU1	PU2	PE
**Initial**				
**Mass of 1 larvae [g]**	0.098 ± 0.001	0.099 ± 0.001	0.099 ± 0.001	0.085 ± 0.002
**Length of 1 larvae [cm]**	2.413 ± 0.013	2.397 ± 0.060	2.367 ± 0.003	2.386 ± 0.047
**Final**				
**Mass of 1 larvae [g]**	0.080 ± 0.002 ^b,^*	0.071 ± 0.003 ^a,b,^*	0.073 ± 0.006 ^a,b,^*	0.064 ± 0.001 ^a,^*
**Length of 1 larvae [cm]**	2.262 ± 0.005 ^a,^*	2.173 ± 0.010 ^a,^*	2.264 ± 0.093 ^a^	2.229 ± 0.005 ^a,^*
**Mass of 1 adult [g]**	0.120 ± 0.001 ^b^	0.114 ± 0.008 ^b^	0.089 ± 0.001 ^a^	0.087 ± 0.007 ^a^
**Length of 1 adult [cm]**	1.617 ± 0.017 ^b^	1.450 ± 0.050 ^a^	1.459 ± 0.092 ^a^	1.322 ± 0.006^a^

Significant differences between initial and final values of a given parameter are indicated with * (Student *t*-test, *p* < 0.05). Post hoc Tukey’s test was completed to show significant differences between all the plastics within a given variable (different letters; *p* < 0.05).

**Table 3:** The concentrations of elements measured in plastics samples by means of EDXRF (± SD; n = 3).

%	PS	PU1	PU2	PE
**Al**	-	0.437 ± 0.038	-	0.180 ± 0.029
**Ba**	-	-	-	0.029 ± 0.001
**Br**	0.412 ± 0.019	-	0.001 ± 0.001	-
**Ca**	0.020 ± 0.009	10.110 ± 0.301	-	1.381 ± 0.020
**Cl**	0.017 ± 0.001	0.224 ± 0.022	7.574 ± 0.141	0.017 ± 0.004
**Co**	0.002 ± 0.001	-	-	-
**Cr**	0.003 ± 0.001	0.008 ± 0.001	0.001 ± 0.000	0.004 ± 0.001
**Cu**	0.006 ± 0.000	0.013 ± 0.001	0.001 ± 0.000	0.006 ± 0.001
**Fe**	0.008 ± 0.002	0.021 ± 0.001	0.001 ± 0.000	0.006 ± 0.001
**Hf**	0.010 ± 0.001	-	-	0.012 ± 0.000
**K**	0.009 ± 0.005	-	0.004 ± 0.002	0.005 ± 0.001
**Mn**	0.003 ± 0.001	0.007 ± 0.001	-	0.002 ± 0.000
**Ni**	-	0.001 ± 0.001	-	-
**P**	-	-	1.379 ± 0.035	-
**S**	0.023 ± 0.002	0.008 ± 0.005	-	0.012 ± 0.003
**Si**	0.049 ± 0.005	0.358 ± 0.027	0.072 ± 0.004	0.029 ± 0.004
**Sn**	-	0.070 ± 0.000	-	-
**Ti**	-	-	-	0.004 ± 0.000
**Zn**	0.003 ± 0.001	0.003 ± 0.000	-	0.007 ± 0.000

**Table 4:** Plastic consumption rates calculated on the basis of literature data and from this publication.

Plastic	Consumption Rate [µg·day^−1^·larvae^−^^1^]	Literature
Polystyrene (PS)	*T. molitor* from Guangzhou: 268.3 *T. molitor* from Tai’an: 160.5 *T. molitor* from Shenzhen: 239.9	[19]
	119.9	[20]
	*T. molitor,* PS alone: 243.0 *T. molitor,* PS + wheat bran: 332.3 *T. obscurus,* PS alone: 324.4 *T. obscurus,* PS + corn flour: 392.4	[40]
	PS alone: 118.0–222.0 PS + soy protein: 491.0 PS + wheat bran: 441.0	[46]
	PS alone: 148.4 PS + wheat bran: 255.2	[47]
	77.4 ^a^ 174.7 ^b^	This research ^a^
Polyurethane (PU)	PU1: 73.9 ^a^ 168.7 ^b^ PU2: 87.5 ^a^ 158.9 ^b^	This research ^a^
Polyethylene (PE)	*T. molitor* from Guangzhou: 172.2 *T. molitor* from Tai’an: 102.7 *T. molitor* from Shenzhen: 138.6	[19]
	PE alone: 226.6 PE + wheat bran: 286.5	[47]
	86.7 ^a^ 122.4 ^b^	This research ^a^

a—calculated from the weight loss data of the given plastic on the 30th day of the experiment (Figure 3). b—calculated from the weight loss data of the given plastic on the 10th day of the experiment (Figure 3).

## Data Availability

Data may be provided upon request to the corresponding author.

## Supplementary Materials

The following are available online at https://www.mdpi.com/article/10.3390/polym13203508/s1, Figure S1: Element contents in *T. molitor* larvae after the experiment, Video S1: Time-lapse movie of the process of plastics waste entomoremediation by *Tenebrio molitor*.
