# Supplementary figures and images for: Biodegradation of Different Types of Plastics by Tenebrio molitor Insect
Source: Polymers (Basel). 2021 Oct 13;13(20):3508. doi: 10.3390/polym13203508 (PMC8537651; doi:10.3390/polym13203508)

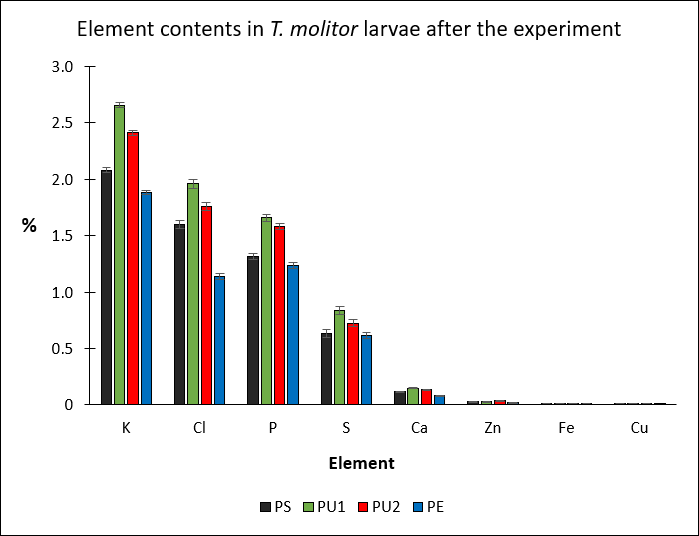

Supplement: Supplementary file 1 [file polymers-13-03508-s001.zip › Figure S1.tif]
